# Combined targeting of SET and tyrosine kinases provides an effective therapeutic approach in human T-cell acute lymphoblastic leukemia

**DOI:** 10.18632/oncotarget.12394

**Published:** 2016-10-01

**Authors:** Nameeta P. Richard, Raffaella Pippa, Megan M. Cleary, Alka Puri, Deanne Tibbitts, Shawn Mahmood, Dale J. Christensen, Sophia Jeng, Shannon McWeeney, A. Thomas Look, Bill H. Chang, Jeffrey W. Tyner, Michael P. Vitek, María D. Odero, Rosalie Sears, Anupriya Agarwal

**Affiliations:** ^1^ Randall Children's Hospital at Legacy Emanuel, Children's Cancer and Blood Disorders Program, Portland, OR USA-97227; ^2^ Division of Oncology, Center for Applied Medical Research (CIMA), University of Navarra, Pio XII-55 Pamplona, Spain-31008; ^3^ Division of Hematology and Medical Oncology, Oregon Health and Science University, Portland, OR, USA-97239; ^4^ Department of Molecular and Medical Genetics, Oregon Health and Science University, Portland, OR USA-97239; ^5^ Research and Development, Oncotide Pharmaceuticals, Research Triangle Park, NC USA 27710; ^6^ Division of Bioinformatics and Computational Biology, Department of Medical Informatics and Clinical Epidemiology, Oregon Health and Science University, Portland, OR, 97239, USA; ^7^ Division of Pediatric Hematology Oncology, Oregon Health and Science University, Portland, OR, USA-97239; ^8^ Dana-Farber Cancer Institute, Harvard Cancer Center, Boston, MA, USA 02215; ^9^ Department of Cell and Developmental Biology, Oregon Health and Science University, Portland, OR, USA-97239; ^10^ Knight Cancer Institute, Oregon Health and Science University, Portland, OR, USA-97239; ^11^ Spyryx Biosciences, Durham, NC USA-27713

**Keywords:** T-ALL, c-MYC, SET, PP2A, tyrosine kinases

## Abstract

Recent evidence suggests that inhibition of protein phosphatase 2A (PP2A) tumor suppressor activity via the SET oncoprotein contributes to the pathogenesis of various cancers. Here we demonstrate that both SET and c-MYC expression are frequently elevated in T-ALL cell lines and primary samples compared to healthy T cells. Treatment of T-ALL cells with the SET antagonist OP449 restored the activity of PP2A and reduced SET interaction with the PP2A catalytic subunit, resulting in a decrease in cell viability and c-MYC expression in a dose-dependent manner. Since a tight balance between phosphatases and kinases is required for the growth of both normal and malignant cells, we sought to identify a kinase inhibitor that would synergize with SET antagonism. We tested various T-ALL cell lines against a small-molecule inhibitor screen of 66 compounds targeting two-thirds of the tyrosine kinome and found that combined treatment of T-ALL cells with dovitinib, an orally active multi-targeted small-molecule receptor tyrosine kinase inhibitor, and OP449 synergistically reduced the viability of all tested T-ALL cell lines. Mechanistically, combined treatment with OP449 and dovitinib decreased total and phospho c-MYC levels and reduced ERK1/2, AKT, and p70S6 kinase activity in both NOTCH-dependent and independent T-ALL cell lines. Overall, these results suggest that combined targeting of tyrosine kinases and activation of serine/threonine phosphatases may offer novel therapeutic strategies for the treatment of T-ALL.

## INTRODUCTION

T-cell acute lymphoblastic leukemia (T-ALL) is an aggressive hematopoietic malignancy that represents 15% of pediatric ALL and 25% of adult ALL cases in the United States [1]. The standard treatment for these patients is intensive chemotherapy; however, 25% of children and more than 50% of adults fail first-line therapy [1, 2]. Cytotoxic chemotherapy leads to acute and chronic side effects, including osteoporosis, peripheral neuropathy, risk of secondary malignancy, and infertility [1]. Additionally, the prognosis for refractory or recurrent ALL is dismal, demanding new molecular targets and improved treatment options for these patients. Emerging evidence suggests that therapies targeted towards specific molecular lesions will be more effective and less toxic for cancer patients [3]. Therefore, oncogenes of interest need to be established before pharmacological intervention can be utilized to attack these targets.

Recently, a variety of genetic lesions, including aberrant expression of TAL1, LYL1, and TLX1/HOX11 [4], activating mutations of NOTCH1[5, 6], and increased expression of c-MYC have been implicated in T-ALL pathogenesis [5, 7, 8]. However, many of these lesions are either difficult to target directly or lack broader applicability. For example, despite the critical role of c-MYC in T-ALL, direct targeting of c-MYC is not currently a clinically viable option. Similarly, targeting NOTCH activating mutations using γ-secretase inhibitors (GSIs) is limited to patients harboring those mutations [9]. Increasing evidence suggests that many of these undruggable yet functionally critical targets are controlled by complex phosphorylation events driven by deregulated activity of specific kinases and phosphatases, which may offer an improved option for molecularly targeted therapy. Accordingly, several studies have recently shown that dephosphorylating c-MYC at serine62 through activation of protein phosphatase 2A (PP2A) reduces c-MYC stability. Targeting c-MYC by reactivation of PP2A might thus be an attractive therapeutic strategy in T-ALL [10–12].

PP2A is a serine/threonine phosphatase involved in cellular proliferation, survival, and differentiation [13]. It has been identified as a tumor suppressor [14, 15] that negatively regulates cell cycle progression and pro-survival molecules [10] and represents a novel therapeutic target in cancer [14, 16–22]. PP2A activity is regulated by a number of inhibitor proteins, including SET and cancerous inhibitor of PP2A (CIP2A). PP2A can be inactivated in cancer cells due to increased accumulation of the endogenous SET and CIP2A oncoproteins, which bind to different subunits of PP2A to inhibit PP2A activity [16, 23]. This leads to increased accumulation of PP2A targets, thus contributing to oncogenesis [24]. Furthermore, it has been shown that high levels of SET and CIP2A at diagnosis are biomarkers for cancer progression in leukemia and confer a poor prognosis [25]. Similarly, overexpression of SET binding protein 1 (SETBP1) protects SET from protease cleavage and permits formation of a SETBP1-SET-PP2A complex, which inhibits PP2A phosphatase activity. Therefore, high SETBP1 levels may also confer a poor outcome in cancer through increased activity of SET and subsequent inhibition of PP2A [26].

We and others have shown that pharmacological re-activation of PP2A or silencing of SET inhibits the growth of cancer cells and reduces disease progression *in vivo* in murine models [14, 21, 23, 27–31]. Additionally, we discovered that the apoE-mimetic peptide OP449 (formerly COG449, Oncotide Inc) [32, 33] inhibits SET, resulting in restoration of PP2A tumor suppressor activity in chronic myelogenous leukemia (CML) and acute myelogenous leukemia (AML) [34]. Based on this evidence, we sought to evaluate the role of the SET/PP2A axis as a therapeutic target in T-ALL. We demonstrate that the SET oncoprotein is overexpressed in various T-ALL cell lines that also display high expression of c-MYC. Further, we demonstrate that SET antagonism using OP449 significantly reduces viability in T-ALL cell lines by reducing the interaction of PP2A with SET. As a consequence, PP2A activity is restored, and expression and activity of c-MYC is drastically decreased.

Additionally, there is increasing evidence demonstrating the role of various tyrosine kinases, such as IGF1R [35], TYK2 [36], or FAK [37], in T-ALL pathogenesis. Since decreased phosphatase function and increased kinase activity is a hallmark of cancer progression, we tested whether activating PP2A through SET antagonism, in combination with tyrosine kinase inhibitors, would reduce survival of T-ALL cells. We discovered that combination therapy using dovitinib to target tyrosine kinases and OP449 to reactivate PP2A is more effective in decreasing the viability of T-ALL cells than either compound alone, thus offering a potential new treatment strategy for T-ALL patients.

## RESULTS

### SET and c-MYC are overexpressed in T-ALL cells compared to T lymphocytes

The overexpression of c-MYC, a well-known PP2A target, has been previously demonstrated in T-ALL [5, 8, 11]. We and others have shown that SET and CIP2A, two oncogenic inhibitors of PP2A, are overexpressed in various cancers, including hematopoietic malignancies [25] and breast cancer [23, 32]. The CIP2A/c-MYC link has been previously reported [38], where CIP2A binds the scaffold subunit of PP2A and prevents c-MYC dephosphorylation at S62, consequently stabilizing c-MYC [11, 38].

**Figure 1:**
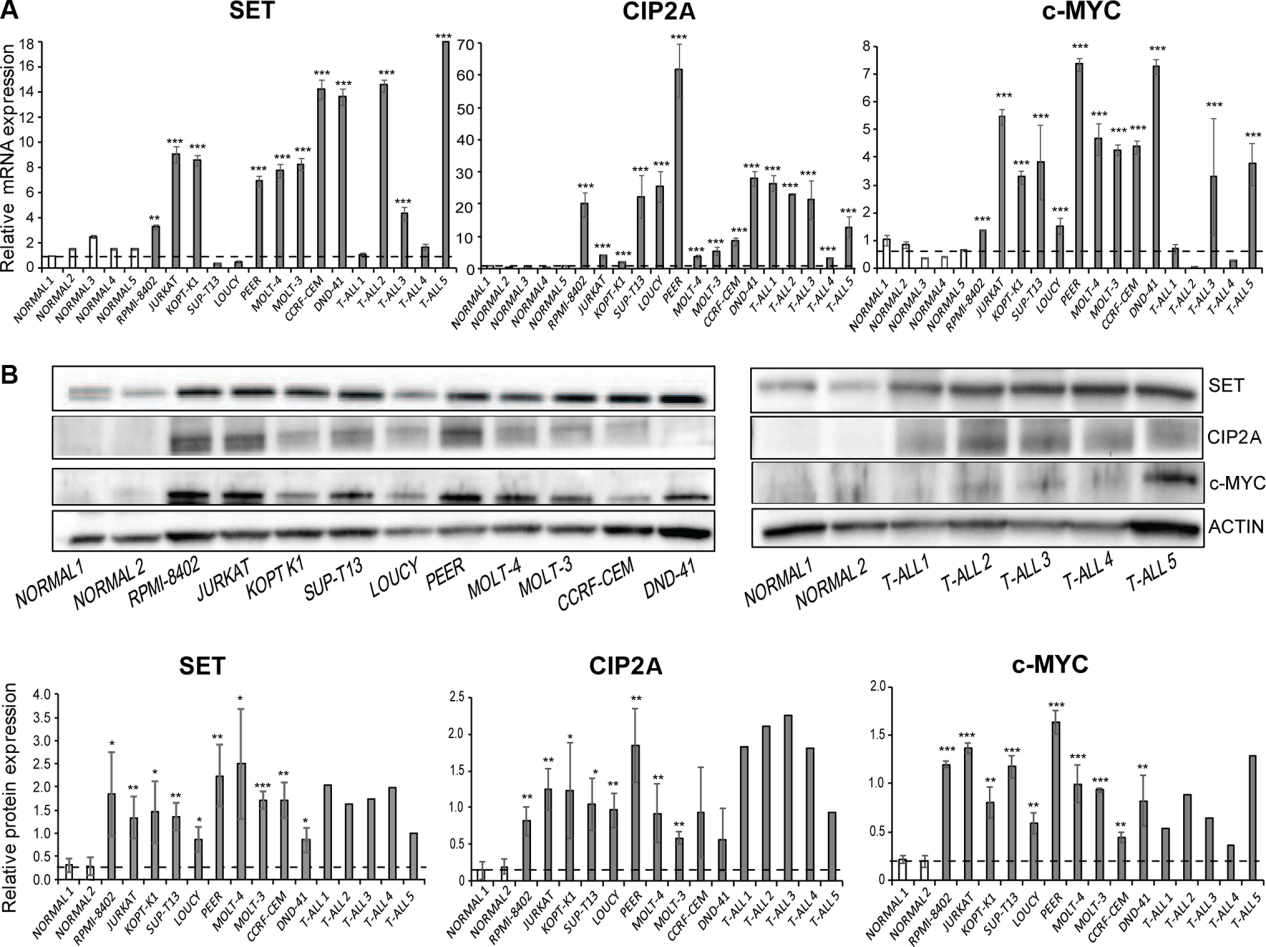
SET and c-MYC are overexpressed in T-ALL cells (**A**) SET, CIP2A, and c-MYC mRNA expression levels in T-ALL cell lines, primary T-ALL samples, and normal CD3^+^ cells were tested by quantitative RT-PCR. (**B**) SET, CIP2A, and c-MYC protein levels were tested by immunoblotting in T-ALL cell lines, primary T-ALL samples, and normal CD3^+^ cells. A representative blot is shown from three independent experiments for cell lines and from two independent analyses from primary T-ALL samples. Protein levels were quantified by performing densitometric analysis of protein expression in each of the indicated samples and normalizing with total actin levels. The dashed horizontal line represents mean of healthy controls used for the analysis. The data represents mean+/−SEM from three independent experiments. *p < 0.05, **p < 0.01, ***p < 0.001.

Regarding SET and c-MYC, we have recently reported that c-MYC plays an important role in the regulation of SET transcription, and correlation analysis showed that SET expression associates with c-MYC in AML patients [39]. To evaluate whether the expression of c-MYC in T-ALL is regulated by the PP2A axis, we first interrogated the expression of c-MYC, SET, CIP2A, and SETBP1 [26] by quantitative RT-PCR (qRT-PCR) in multiple cell lines and primary samples derived from T-ALL patients, compared to control T cells derived from healthy individuals. We found that c-MYC mRNA levels were 2- to 7-fold higher in T-ALL cell lines and some primary T-ALL samples compared to control T cells purified from healthy samples (Figure 1A, Supplementary Table S1). Further, both SET and CIP2A mRNA levels were increased up to 16-fold and 60-fold, respectively, in T-ALL cells compared to control cells. Consistent with higher mRNA levels, we observed increased c-MYC, SET, and CIP2A protein levels in T-ALL cell lines compared to normal T cells. Accordingly, SET expression was also high in primary T-ALL samples compared to normal BM, peripheral blood, and thymus cells as evident from the analysis of three independent databases (Supplementary Figure S1). Notably SETBP1 expression was increased in T-ALL cell lines and in few primary T-ALL cells compared to normal T cells (Supplementary Figure S2). The expression of wild-type NOTCH, as in LOUCY and JURKAT cells [40], or mutated NOTCH, as in RPMI-8402 and MOLT-4 cells [40, 41], did not differentially affect this upregulation of c-MYC, SET, CIP2A, and SETBP1 (Figure 1B). Increased expression of PP2A activity regulators SET, CIP2A, and SETBP1 across various T-ALL cell lines and primary samples strongly suggests a role for these PP2A activity regulators and the PP2A axis in T-ALL. Previous studies have shown that PP2A targets c-MYC and dephosphorylates the stabilizing residue serine 62, leading to c-MYC ubiquitination and its subsequent degradation by the proteasome [11, 42]. One possibility for the increase in c-MYC expression in T-ALL could be that increased SET and CIP2A expression mediates inhibition of PP2A activity, leading to stabilization of the c-MYC protein. Our results indicate that targeting the PP2A axis may be a potential strategy in both NOTCH-dependent and independent T-ALL cells.

### SET antagonism promotes PP2A activity and inhibits T-ALL cell growth

**Figure 2:**
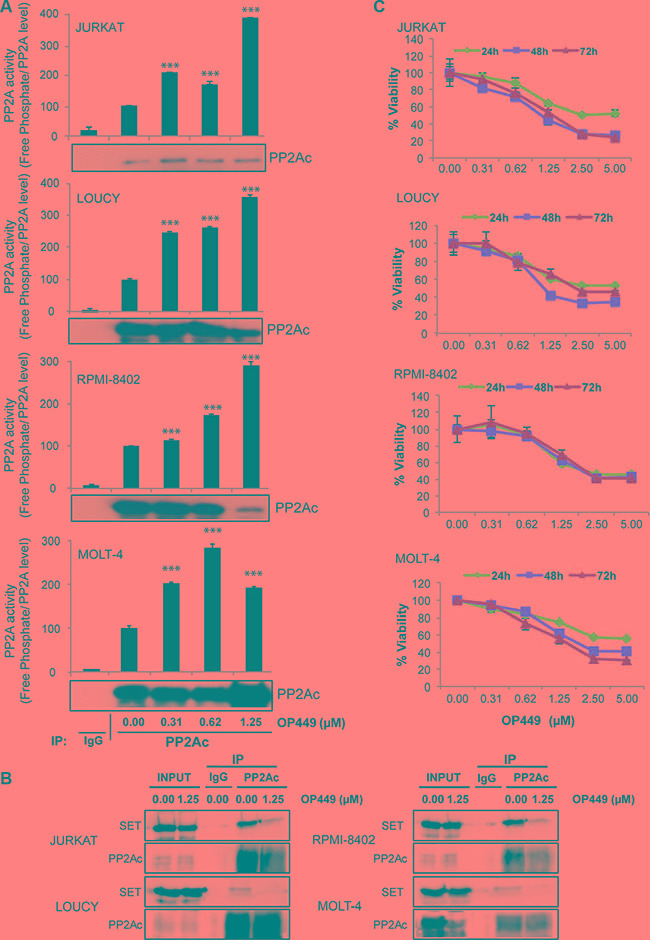
OP449 inhibits growth of T-ALL cells by reactivating PP2A (**A**) Indicated T-ALL cell lines were exposed to graded concentrations of OP449 for 24 hrs and free phosphate levels were measured. Results are presented as free phosphate levels normalized to the total amount of immunoprecipitated PP2Ac protein as detected by western blot analysis ± standard deviation. PP2Ac levels were quantified by densitometric analysis of immunoprecipitated protein in each sample. ***denotes *p* < 0.001 compared to untreated control. (**B**) Effect of OP449 on the interaction of SET/PP2A in T-ALL cells. Co-immunoprecipitation experiments were performed with T-ALL cell lines treated with vehicle or 1.25 μM OP449 for 4 hrs using anti-PP2Ac antibody or non-specific IgGs (used as a control) . Then, the immunoprecipitates were separated by SDS PAGE, transferred onto PVDF and analyzed for the presence of PP2Ac and its inhibitor SET. (**C**) Effect of OP449 on the growth of T-ALL cells. T-ALL cell lines were cultured in graded concentrations of OP449 and cell viability was measured at 24, 48, and 72 hrs by colorimetric MTS assay in three independent experiments. Results are graphed as the mean percent viability relative to untreated cells ± standard deviation.

Recently, we showed that the peptide-based drug OP449 selectively binds to SET and restores the activity of PP2A [21, 32], causing decreased cell viability in CML and AML [34]. Consistent with this, we observed that the treatment of T-ALL cell lines with OP449 increased PP2A activity in a dose-dependent manner. JURKAT, LOUCY, RPMI-8402, and MOLT-4 cells exhibited a 3- to 4-fold increase in PP2A activity after being treated with 0.62–1.25 μM OP449 (Figure 2A, *p* < 0.001). In addition, treatment with OP449 reduced the association of SET with the PP2A catalytic subunit (PP2Ac) (Figure 2B), suggesting that OP449 may increase PP2A activity by reducing PP2A's association with SET. This increase in PP2A activity with OP449 treatment correlated with a decrease in cell viability in a dose-dependent manner, with an IC_50_ value of 1.25 μM at 72 hrs across all of these T-ALL cell lines (Figure 2B, *p* < 0.001 compared to untreated control), together with a dose-dependent increase in cellular apoptosis as reflected by annexin V staining (Supplementary Figure S3). Taken together, these data suggest that OP449 inhibits growth of both NOTCH-dependent and independent T-ALL cells by antagonizing SET inhibition of PP2A.

### Dovitinib, a multi-kinase inhibitor, decreases T-ALL cell growth and synergistically inhibits T-ALL growth with OP449

**Figure 3:**
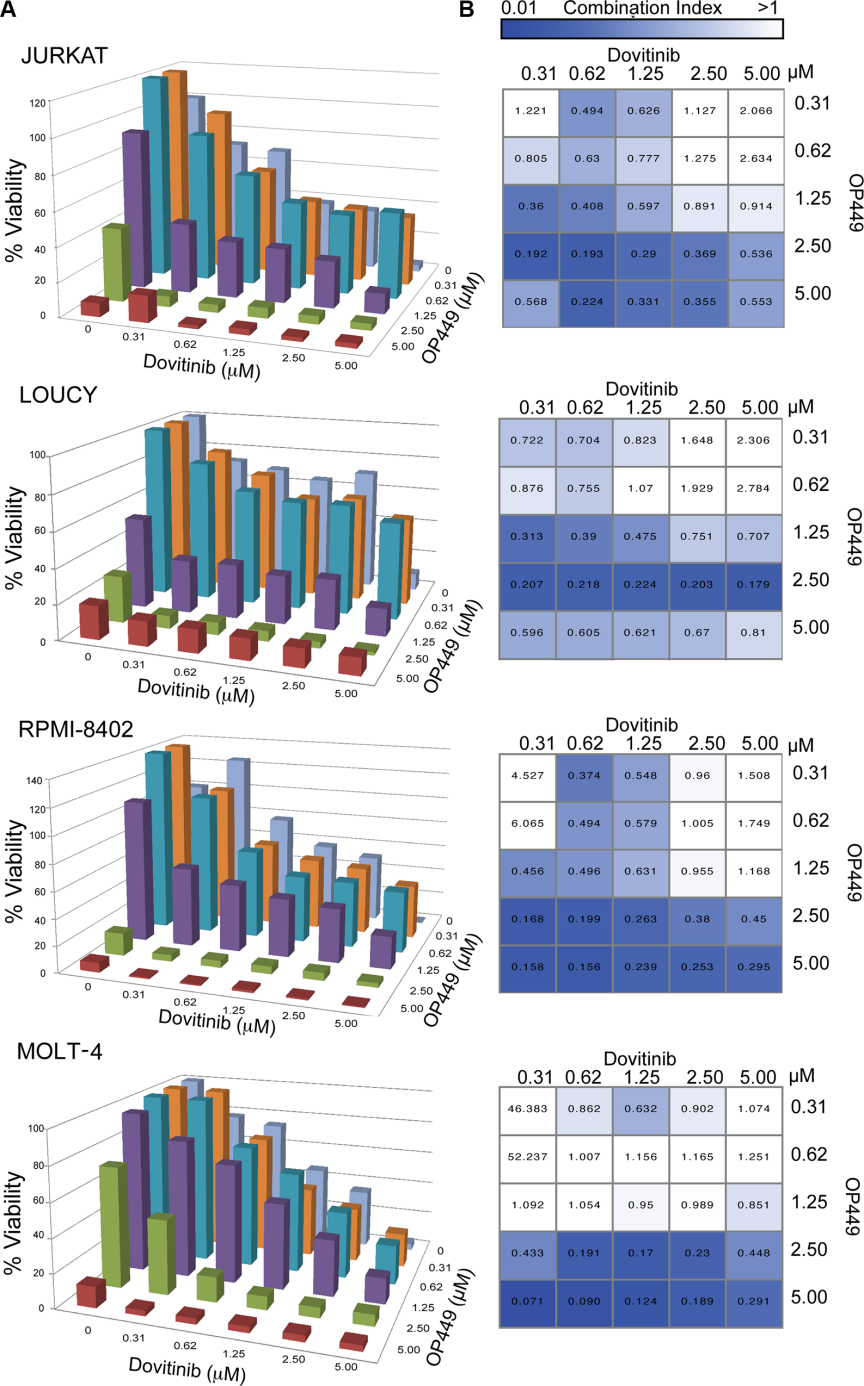
Combination of OP449 and the multi-targeted tyrosine kinase inhibitor dovitinib synergistically inhibits T-ALL growth synergistically (**A**) Combined treatment with OP449 or dovitinib on T-ALL cell lines (Additional cell lines are in Supplementary Figure S4). Cells were incubated for 72 hrs alone or in combination and cell viability was measured by standard MTS assay. (**B**) Heatmap of combination indices calculated using Calcusyn corresponding to (A), where values less than one were considered synergistic.

Combined targeting of tyrosine kinases and activating phosphatases has been explored as a therapeutic approach for targeting cancer cells [43, 44]. We therefore attempted to identify a kinase inhibitor that would be synergistic with SET antagonism. We tested 9 T-ALL cell lines derived from pediatric T-ALL patients on a well-characterized small-molecule inhibitor screen comprising 66 compounds targeting various kinases [45]. We identified several kinase inhibitors targeting the PI3K/AKT, MAPK, and JAK pathways, as well as pan-tyrosine kinase inhibitors, that uniformly inhibited the growth of T-ALL cells. We tested the synergistic effect of OP449 with at least one agent from each subclass (Supplementary Table S2) and identified the most significant effect with dovitinib[46, 47]. Dovitinib is an orally active small molecule targeting tyrosine kinases including FGFR1/3, VEGFR1-4, FLT-3, and c-KIT [48], and is particularly effective in decreasing growth of various T-ALL cell lines. At 72 hrs, IC_50_ values ranged from 0.5–1.2 μM for both NOTCH-dependent (RPMI-8402 and MOLT-4) and independent cell lines (LOUCY and JURKAT) (Figure 3A). To determine whether combined targeting of relevant tyrosine kinases and phosphatases would reduce T-ALL cell growth synergistically, we used a matrix of various concentrations of dovitinib and OP449 to assess their effects on the growth of 9 T-ALL cell lines. Most of the tested T-ALL cell lines showed a synergistic reduction in cell growth upon treatment with dovitinib and OP449, as determined by calculation of a combination index (CI) (Figure 3B, Supplementary Figure S4B). For example, treatment of LOUCY cells with 1.25 μM OP449 or 0.312 μM dovitinib alone reduced cell viability by 48% and 28% (Figure 3A), respectively, while combined treatment with these concentrations synergistically reduced cell viability by 60% (CI value 0.313, Figure 3A, 3B). Similar trends were observed for most T-ALL cell lines tested (Figure 3, Supplementary Figure S4). These results demonstrate that dovitinib offers an effective therapeutic combination with OP449 for inhibiting T-ALL cell growth *in vitro*.

### Combined treatment with OP449 and dovitinib reduces c-MYC and kinase activity

**Figure 4:**
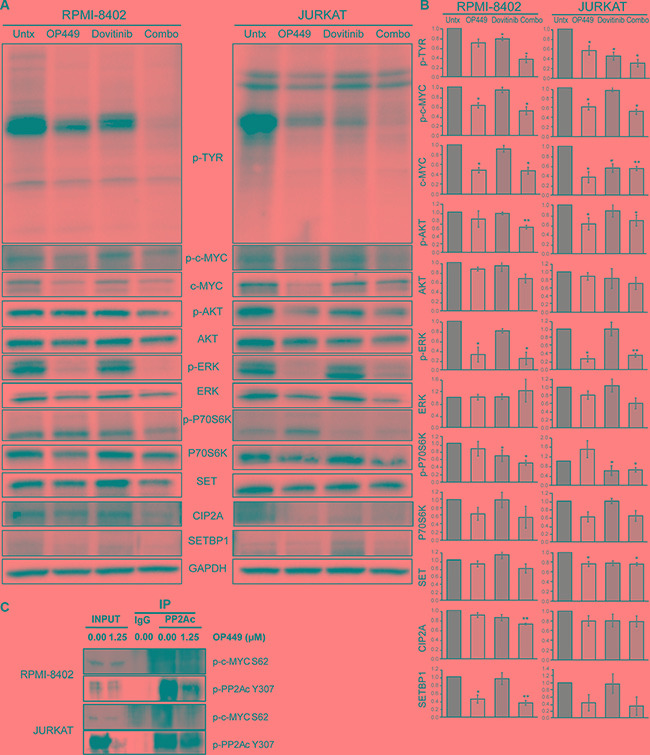
OP449 and dovitinib synergistically inhibit downstream signaling (**A**) RPMI-8402 and JURKAT cell lines were incubated 24 hrs in the presence of 1 μM OP449, 1 μM dovitinib, or combination. Cells were lysed and immunoblotted with the indicated antibodies. A representative blot is shown. (**B**) Protein levels were quantified by performing densitometric analysis for each of the indicated proteins for various treatments and normalizing to GAPDH controls. For pan-phosphotyrosine (p-TYR) blot, all distinct bands were quantitated separately and then consolidated into one graph. The relative protein expression was represented as fold changes over vehicle-treated control. The data represent mean+/−SEM from three independent experiments. *p < 0.05, **p < 0.01 (**C**) PP2Ac co-immunoprecipitation experiments in RPMI-8402 and JURKAT cell lines treated with vehicle or 1.25 μM OP449 for 8 hrs. Then, the immunoprecipitates were analyzed for the presence of phospho-PP2A Y307 and phospho-c-MYC S62.

To investigate the mechanism by which dovitinib and OP449 decrease cell viability in T-ALL, we tested the effect of these treatments on c-MYC levels and on phosphorylation of kinases previously shown to have activity in T-ALL cell lines expressing wild-type NOTCH (JURKAT) or mutated NOTCH (RPMI-8402). The treatment of RPMI-8402 and JURKAT cells with OP449 reduced total c-MYC levels and phosphorylation of c-MYC at serine 62 (Figure 4A, 4B). Since previous studies have shown that decreased phosphorylation of the PP2Ac subunit at Y307 is correlated with increased PP2A activity [18] and that decreased phosphorylation of c-MYC at serine 62 reduces c-MYC stability [10, 11], we investigated whether phosphorylation of these proteins is reduced in T-ALL cell lines upon treatment with OP449. We found a decrease in both PP2Ac Y307 phosphorylation and an association of phospho-S62 c-Myc with PP2Ac (Figure 4C), suggesting that SET antagonism reduced c-MYC stability by promoting PP2A activity. Next, to determine whether SET antagonism influences c-MYC activity, we tested the effect of OP449 treatment on direct targets of c-MYC, such as transcription factor E2F2 and proteins involved in ribosome biogenesis, including 5sRNA and nucleolin (Supplementary Figure S5) [49]. Our data indeed showed that OP449 treatment reduced the expression of c-MYC targets E2F2, 5sRNA, and nucleolin, corroborating our hypothesis that SET inhibition has a negative effect on the activity of c-MYC. Additionally, OP449 treatment led to a reduction in SET, CIP2A, and SETBP1 levels (Figure 4A, 4B), possibly due to a decrease in c-MYC activity and increase in PP2A activity [39, 50].

**Figure 5:**
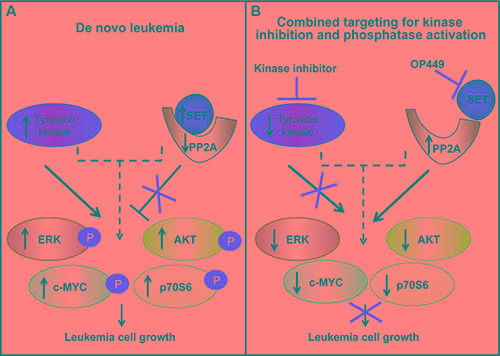
A model showing that combining phosphatase reactivation using SET antagonism with tyrosine kinase inhibition blocks the growth of T-ALL cells (**A**) SET is overexpressed in leukemic cells, which leads to decreased PP2A phosphatase activity, and loss of deactivation of downstream kinases. Leukemia cells have increased tyrosine kinase activity that promotes downstream signaling. (**B**) Combined targeting of SET and activated kinases leads to a more pronounced inhibition of leukemic cell growth in T-ALL.

Further, we observed differential sensitivity of OP449 and dovitinib in inhibiting downstream kinase activity (Figure 4A, 4B). Treatment with OP449 reduced phosphorylation of ERK1/2 in RPMI-8402 cells and phosphorylation of ERK1/2 and AKT in JURKAT cells. Treatment with dovitinib diminished phosphorylation of p70S6 kinase and pan-phosphotyrosine activity. Interestingly, combined treatment with OP449 and dovitinib induced both OP449- and dovitinib-mediated effects and blocked the phosphorylation of c-MYC, AKT, ERK1/2, p70S6, and pan-phosphotyrosine activity in both NOTCH-dependent and independent T-ALL cell lines. These results offer a novel therapeutic approach where the combined targeting of T-ALL cells with a multi-kinase inhibitor and PP2A activator synergistically reduces growth by inhibiting multiple oncogenic pathways in both NOTCH-dependent and independent T-ALL cells (Figure 5).

## DISCUSSION

The outcome of pediatric cases of ALL has improved tremendously over the last 25 years [51], and dose intensification of cytotoxic drugs now appears to be optimized; however, 25% of children and more than 50% of adults fail first-line therapy [1, 2], leaving a gap in survival that current therapies fail to fill. Additionally, patients cured of T-ALL through standard treatment suffer numerous side effects that can lead to poor quality of life. Therefore, a new approach for novel treatment options is desperately needed.

Half of T-ALL cases harbor activating mutations in NOTCH [52, 53]. c-MYC is a direct transcriptional target of NOTCH; however, c-MYC protein is elevated in T-ALL cells regardless of NOTCH dependency [54], suggesting a broader application of interfering with c-MYC signaling than targeting NOTCH. Recent studies have identified that c-MYC can be destabilized by increasing the activity of the serine/threonine phosphatase PP2A [10–12]. PP2A is a tumor suppressor [14, 15] that can be inactivated in cancer cells due to increased accumulation of the SET and CIP2A oncoproteins [16, 23, 25]. This prevents the downregulation of PP2A targets such as c-MYC, thus contributing to oncogenesis [23]. Targeting the SET/PP2A or CIP2A/PP2A axes might successfully destabilize c-MYC and disrupt aberrant cellular function. Although the specific mechanism by which CIP2A inhibits PP2A is believed to be through direct binding to the scaffold subunit of PP2A, the details of this interaction are still unknown, and CIP2A inhibitors have yet to be designed. Therefore, in this work, we focused on SET and sought to determine the role of the SET/PP2A axis in T-ALL by using a SET antagonist, OP449, which has been previously shown to be effective against CLL, non-Hodgkin's lymphoma, CML, and AML cells *ex vivo* [32, 34]. Here we report a novel role of SET antagonism in T-ALL and show that combined targeting of tyrosine kinases and activation of PP2A may offer an effective and novel therapeutic strategy for the treatment of NOTCH-dependent and independent T-ALL.

We found that SET, CIP2A, and c-MYC mRNA and protein levels were frequently elevated in both NOTCH-dependent and independent cell lines and primary T-ALL samples compared to normal T cells, which is consistent with our recent finding that c-MYC activates the transcription of SET and possibly CIP2A in AML [39]. Next, we investigated the *in vitro* efficacy of the SET inhibitor OP449 on T-ALL cell lines and demonstrated a dose-dependent increase in PP2A activity and a decrease in cell viability and survival, supporting previous findings regarding the oncogenic properties of SET in hematopoietic malignancies [16, 18, 32, 34]. We observed that treatment with OP449 reduced total c-MYC levels and serine 62 phosphorylation of c-MYC. Because phosphorylation at serine 62 stabilizes c-MYC, these results suggested that PP2A reactivation via SET antagonism regulates c-MYC stability, consistent with previous studies showing the role of PP2A in stabilization of c-MYC [11]. Additionally, OP449 treatment reduced c-MYC activity as evidenced by decreased expression of direct c-MYC targets E2F2, 5sRNA, and nucleolin. OP449 treatment also led to a reduction in CIP2A and SETBP1 levels. The reduction in CIP2A levels may be due to a decrease in c-MYC activity, which is known to regulate CIP2A expression [50]. SETBP1 may be destabilized due to an increase in PP2A activity and reduction in c-MYC and SET levels, as we previously reported in AML [39]. Overall, the increase in free phosphate together with decreased binding of PP2Ac to SET, decreased phosphorylation of PP2Ac at Y307, and decreased c-MYC stability with OP449 treatment, indicates reactivation of PP2A and the specificity of the compound to antagonize the SET oncoprotein.

Since increasing evidence has demonstrated the role of various tyrosine kinases such as IGF1R [35], TYK2 [36], or FAK kinases [37] in T-ALL pathogenesis, we identified a kinase inhibitor to synergize with SET antagonism and enhance the potency of OP449. This combined strategy of phosphatase activation with OP449 and kinase inhibition with the multi-tyrosine kinase inhibitor dovitinib demonstrated synergy in reducing cell viability *in vitro*. OP449 treatment itself reduced phosphorylation of ERK1/2 and AKT, while dovitinib treatment diminished phosphorylation of p70S6 kinase and pan-phosphotyrosine activity. Notably, combined targeting decreased phosphorylation of c-MYC, AKT, ERK1/2, p70S6, and pan-phosphotyrosine levels. Our data suggests that dovitinib might be effective in T-ALL because it has the ability to inhibit multiple kinases together, as evidenced by reduced pan-phosphotyrosine kinase activity. Dovitinib is known to inhibit several kinases, including FLT3, c-KIT, FGFR1/3, and VEGFR1-4 [46, 48]. However, in our small-molecule inhibitor screen with T-ALL cells, we have not seen consistent activity of FLT-3/c-KIT inhibitor (sunitinib), FGFR1/3 inhibitor (ponatinib), or VEGFR inhibitor (sorafenib, AMG 706) [45], suggesting further investigation needed to identify the specific targets for dovitinib in T-ALL.

Our data suggest that activation of the serine/threonine phosphatase PP2A by OP449 treatment not only reduces serine/threonine phosphorylation of c-MYC, AKT, and p70S6, but also reduces the activity of ERK1/2, which is phosphorylated at both threonine and tyrosine residues, and pan-phosphotyrosine activity. This is consistent with previous studies showing that increased PP2A activity is associated with increased tyrosine phosphatase activity of SHP-1, which in turn dephosphorylates tyrosine kinases [24], and that SHP-1 can itself induce tyrosine dephosphorylation of PP2A [55]. Accordingly, our data show that OP449 treatment reduced tyrosine phosphorylation of PP2Ac and associated pan-phosphotyrosine activity. Additionally, both NOTCH-dependent and independent cell lines responded to treatment with OP449 and dovitinib, suggesting that SET antagonism in combination with this kinase inhibitor will be beneficial irrespective of NOTCH status in T-ALL cells. Further, NOTCH activating mutations can be therapeutically targeted with γ-secretase inhibitors (GSIs); however, GSIs may reduce PP2A activity, leading to reactivation of many oncogenic proteins such as c-MYC [9, 56]. Our data suggests that targeting the SET/PP2A axis should be beneficial for reactivation of PP2A, thus blocking downstream signaling in T-ALL with activated NOTCH. Our results are in accordance with recent studies suggesting the antipsychotic drug perphenazine (PPZ) could be repurposed for activating PP2A in T-ALL cells [57].

Overall, our study offers additional insight suggesting that antagonizing SET in combination with tyrosine kinase inhibition will be beneficial for T-ALL patients (Figure 5). Our previous work has shown that antagonism of SET does not cause any toxicity in an *in vivo* mouse model[34], which may help in reducing some of the toxicity of standard-of-care chemotherapy drugs. These results support ongoing efforts for the development of small molecules targeting the SET/PP2A axis [58], which may provide better outcomes not only for larger subsets of T-ALL patients, but also for other cancer types.

## MATERIALS AND METHODS

### Cell culture and normal T cells

Certified CCRF-CEM, JURKAT, LOUCY, MOLT-4, PEER, RPMI-8402, DND41, SUPT13 and KOPTK1 cell lines were generously provided by Dr. A. Thomas Look at Dana Farber Cancer Institute. MOLT-3 cell lines were purchased from ATCC (CRL-1552, Manassas, Va). All cell lines were maintained in RPMI-1640 medium supplemented with 2 mM L-glutamine, 10% FBS, 100 U/ml penicillin, 100 μg/mL streptomycin. LOUCY cell line was grown in RPMI-1640 with 20% FBS. All cell lines were cultured at 37°C in 5% CO2. Fresh peripheral blood was collected from consented donors in accordance with OHSU IRB guidelines. T cells were isolated from fresh PBMCs by magnetic bead separation using the EasySep Human CD3 Positive Selection Kit (Stem Cell Technologies) according to manufacturer's instructions. T cells were counted and assessed for viability by trypan blue exclusion, which was always > 95%.

### Cell viability assay and inhibitor screen

OP449 was obtained from Oncotide Pharmaceuticals (Research Triangle Park, NC) (molecular weight 9223 g/mol) and was reconstituted in PBS as a 10 mM stock and prepared as previously described [34]. Dovitinib was purchased from Selleckchem (S1018, Houston, TX) and reconstituted with DMSO as 20 mM stock for *in vitro* studies. Sensitivity to OP449 and dovitinib was tested using a concentration gradient in a 96 well plate, seeding 5000 cells per well. Cells were cultured in their respective media for 72 hrs. The cytotoxic effect of drugs was assessed by Cell Titer 96 Aqueous One solution cell proliferation assay (MTS) (Promega, Madison, WI) and a BioTek Synergy 2 plate reader (Winooski, VT). The IC_50_ for each compound was calculated for each cell line. Combination indices (CI) were calculated using Calcusyn software. We performed a small molecule inhibitor screen comprised of 66 compounds targeting two-thirds of the tyrosine kinome as described previously [45].

### Immunoblotting

T-ALL cell lines were cultured in the presence or absence of 1 μM OP449, 1 μM dovitinib, or combination. Following the indicated drug exposure time, cells were washed in PBS and lysed in 50 μL lysis buffer (9803, Cell Signaling Technology, Boston, MA) supplemented with complete protease inhibitor and phosphatase inhibitor cocktail-2 (Sigma-Aldrich, St. Louis, MO). Equal amounts of protein were fractionated on 4–15% Tris-glycine polyacrylamide gels (Bio-Rad, Hercules, CA), transferred to PVDF membranes and probed with antibodies to SETBP1 (98222) and p-cMYC (78318) from AbCam, San Francisco, CA; c-MYC (764) from Santa Cruz, Dallas, TX; p-AKT (4060), AKT (9272), p-ERK1/2 (9101), ERK1/2 (4695), p-p70S6K (9234), and p70S6K (2708) from Cell Signaling, Danvers, MA; p-Tyr (05-321), actin (C4), and PP2A-C (05-421) from Millipore, Temecula, CA; SET (A302-261a) from Bethyl, Montgomery, TX; CIP2A (a gift from Jukka Westermarck, Finnish Cancer Institute, Turku Centre for Biotechnology, Turku, Finland), p-PP2A-Y307 (1155-1) from Epitomics, Cambridge, MA; and GAPDH (AM4300) from ThermoFisher, Carlsbad, CA.

### PP2A assay

PP2A activity was determined as previously described [14, 34] using the commercially available PP2A Immunoprecipitation Phosphatase Assay Kit (17-313, Upstate Biotechnology). Briefly, protein lysates were prepared in 20 mM imidazole-HCl, 2 mM EDTA, 2 mM EGTA, pH 7.0 with 10 μg/ml each of aprotinin leupeptin and pepstatin, 1 mM benzamidine, 1mM PMSF and phosphatase inhibitors tablets (Roche, Madison, WI). 50 μg of protein was immunoprecipitated with 2 μg of anti-PP2A antibody (1D6, Upstate Biotechnology) and 50 μL of protein-A-agarose beads for 2 hrs at 4°C. Beads were washed extensively with lysis buffer, then with Ser/Thr assay buffer. Beads were then used in the phosphatase reaction for measuring dephosphorylation of the phosphopeptide (K-R-pT-IR-R) according to the manufacturer's protocol using malachite green phosphate detection solution. The level of free phosphate was normalized to total amount of PP2Ac immunoprecipitated as measured by densitometry analysis of immunoblots.

### Immunoprecipitation

OP449 or vehicle-treated cell pellets were lysed in 300 μl of co-IP buffer (PBS containing 0.5% Triton X-100, 1 mM EDTA, 100 μM sodium orthovanadate, 0.25 mM PMSF and complete protease inhibitor mixture, Roche). After centrifugation, protein samples were quantified by the BCA method and incubated over night at 4°C with 4 μg of PP2Ac antibody followed by two hours incubation with protein A/G- agarose beads (Invitrogen). The immunocomplexes were extensively washed with co-IP buffer, subsequently eluted with 25 μl of Laemmli buffer and boiled at 100ºC for Western blot analysis. IP control samples were incubated with irrelevant IgG.

### Quantitative PCR

Total RNA was extracted using an RNeasy Kit (QIAGEN, Valencia, CA) and cDNA was synthesized using Oligo(dT)-primers with a SuperScript First Strand Synthesis kit (Invitrogen, Carlsbad, CA). cDNA was quantified and normalized using a NanoDrop 1000 spectrophotometer. Quantitative PCR was performed in triplicate using Taqman primer/ probe sets (Applied Biosystems) for c-MYC (Hs00905030_m1), SET (Hs00853870_g1), CIP2A (Hs00405413_m1), and for normalization 18S (Hs99999901_s1) genes. Reactions were carried out using 5ng of cDNA and PerfeCta qPCR FastMix (Quanta Biosciences) on a StepOne Real-Time PC System machine (Applied Biosystems). For E2F2, Nucleolin, and 5sRNA RT-PCR reactions were performed with the QuantStudio 7 Flex System (Thermo Fisher Scientific, Grand Island, NY) using Platinum SYBR Green qPCR SuperMix (Invitrogen) according to manufacturer's instructions. ROX reference dye was used to normalize fluorescent signal between reactions. The following primers: Nucleolin forward: 5′-ACTGACCGGGAAACTGGGTC-3′ and reverse: 5′-TG GCCCAGTCCAAGGTAACT-3′, E2F2 forward: 5′-ACA AGGCCAACAAGAGGCTG-3′ and reverse: 5′-TCAGT CCTGTCGGGCACTTC-3′, 5sRNA forward: 5′-GGCC ATACCACCCTGAACGC-3′ and reverse: 5′-CAGCACC CGGTATTCCCAGG-3′, and for normalization GAPDH forward: 5′-TCCTGCACCACCAACTGCTTAG-3′ and reverse: 5′-GGCATGGACTGTGGTCATGAG-3′ were used.

### SET expression analysis using databases

Oncomine^™^ (Compendia Bioscience, Ann Arbor, MI) was used for examination of putative differential expression and visualization for SET. Specifically, the expression of SET was examined between T-ALL and normal samples in the Andersson and Haferlach datasets. All Oncomine data sets are log-transformed and median-centered per array [59]. In addition, SET expression was examined between T-ALL and thymus samples using GEO's GSE46170 dataset (unpublished). Raw data was downloaded and normalized using quantile normalization and RMA background correction. (Bioconductor's affy package 1.46.1).

### Statistical methods

IC_50_ values were generated using GraphPad Prism software. Combination indices (CI) were calculated using Calcusyn software. A CI value less than 1.0 represents a synergistic drug combination. Statistical comparisons were performed using a two-tailed, unpaired Student's *t*-test when comparing expression between healthy and leukemia samples and two-tailed, paired Student's *t*-test when comparing untreated and treated groups from the same sample. A *p* value less than 0.05 was considered statistically significant. * denotes *p* < 0.05, ** denotes *p* < 0.01, and *** denotes *p* < 0.001. Densitometry analysis was conducted using Image Lab software (Bio-Rad, Hercules, CA).

## SUPPLEMENTARY MATERIALS FIGURES AND TABLES



## References

[1] Pui C-H, Evans WE (2006). Treatment of Acute Lymphoblastic Leukemia. NEJM.

[2] Goldberg JM, Silverman LB, Levy DE, Dalton VK, Gelber RD, Lehmann L, Cohen HJ, Sallan SE, Asselin BL (2003). Childhood T-cell acute lymphoblastic leukemia: the Dana-Farber Cancer Institute acute lymphoblastic leukemia consortium experience. JCO.

[3] Druker BJ, Guilhot F, O'Brien SG, Gathmann I, Kantarjian H, Gattermann N, Deininger MW, Silver RT, Goldman JM, Stone RM, Cervantes F, Hochhaus A, Powell BL (2006). Five-year follow-up of patients receiving imatinib for chronic myeloid leukemia. NEJM.

[4] Van Vlierberghe P, Ferrando A (2012). The molecular basis of T cell acute lymphoblastic leukemia. JCI.

[5] (2014). T-ALL pathogenesis requires a NOTCH1-driven MYC enhancer. Cancer Discov.

[6] Grabher C, von Boehmer H, Look AT (2006). Notch 1 activation in the molecular pathogenesis of T-cell acute lymphoblastic leukaemia. Nat Rev Cancer.

[7] Kaveri D, Kastner P, Dembele D, Nerlov C, Chan S, Kirstetter P (2013). beta-Catenin activation synergizes with Pten loss and Myc overexpression in Notch-independent T-ALL. Blood.

[8] Roderick JE, Tesell J, Shultz LD, Brehm MA, Greiner DL, Harris MH, Silverman LB, Sallan SE, Gutierrez A, Look AT, Qi J, Bradner JE, Kelliher MA (2014). c-Myc inhibition prevents leukemia initiation in mice and impairs the growth of relapsed and induction failure pediatric T-ALL cells. Blood.

[9] Hales EC, Taub JW, Matherly LH (2014). New insights into Notch1 regulation of the PI3K-AKT-mTOR1 signaling axis: targeted therapy of gamma-secretase inhibitor resistant T-cell acute lymphoblastic leukemia. Cell Signal.

[10] Yeh E, Cunningham M, Arnold H, Chasse D, Monteith T, Ivaldi G, Hahn WC, Stukenberg PT, Shenolikar S, Uchida T, Counter CM, Nevins JR, Means AR (2004). A signalling pathway controlling c-Myc degradation that impacts oncogenic transformation of human cells. Nat Cell Bioly.

[11] Arnold HK, Sears RC (2006). Protein phosphatase 2A regulatory subunit B56alpha associates with c-myc and negatively regulates c-myc accumulation. MCB.

[12] Neviani P, Perrotti D (2014). SETting OP449 into the PP2A-activating drug family. Clin Canc Res.

[13] Janssens V, Goris J (2001). Protein phosphatase 2A: a highly regulated family of serine/threonine phosphatases implicated in cell growth and signalling. Biochem J.

[14] Perrotti D, Neviani P (2008). Protein phosphatase 2A (PP2A), a drugable tumor suppressor in Ph1(+) leukemias. Cancer Metast Rev.

[15] Cristobal I, Garcia-Orti L, Cirauqui C, Alonso MM, Calasanz MJ, Odero MD (2011). PP2A impaired activity is a common event in acute myeloid leukemia and its activation by forskolin has a potent anti-leukemic effect. Leukemia.

[16] Cristobal I, Garcia-Orti L, Cirauqui C, Cortes-Lavaud X, Garcia-Sanchez MA, Calasanz MJ, Odero MD (2012). Overexpression of SET is a recurrent event associated with poor outcome and contributes to protein phosphatase 2A inhibition in acute myeloid leukemia. Haematologica.

[17] Neviani P, Santhanam R, Oaks JJ, Eiring AM, Notari M, Blaser BW, Liu S, Trotta R, Muthusamy N, Gambacorti-Passerini C, Druker BJ, Cortes J, Marcucci G (2007). FTY720, a new alternative for treating blast crisis chronic myelogenous leukemia and Philadelphia chromosome-positive acute lymphocytic leukemia. JCI.

[18] Neviani P, Santhanam R, Trotta R, Notari M, Blaser BW, Liu S, Mao H, Chang JS, Galietta A, Uttam A, Roy DC, Valtieri M, Bruner-Klisovic R (2005). The tumor suppressor PP2A is functionally inactivated in blast crisis CML through the inhibitory activity of the BCR/ABL-regulated SET protein. Cancer Cell.

[19] Roberts KG, Smith AM, McDougall F, Carpenter H, Horan M, Neviani P, Powell JA, Thomas D, Guthridge MA, Perrotti D, Sim AT, Ashman LK, Verrills NM (2010). Essential requirement for PP2A inhibition by the oncogenic receptor c-KIT suggests PP2A reactivation as a strategy to treat c-KIT+ cancers. Cancer Res.

[20] Samanta AK, Chakraborty SN, Wang Y, Kantarjian H, Sun X, Hood J, Perrotti D, Arlinghaus RB (2009). Jak2 inhibition deactivates Lyn kinase through the SET-PP2A-SHP1 pathway, causing apoptosis in drug-resistant cells from chronic myelogenous leukemia patients. Oncogene.

[21] Switzer CH, Cheng RY, Vitek TM, Christensen DJ, Wink DA, Vitek MP (2011). Targeting SET/IPP2A oncoprotein functions as a multi-pathway strategy for cancer therapy. Oncogene.

[22] Yang Y, Huang Q, Lu Y, Li X, Huang S (2012). Reactivating PP2A by FTY720 as a novel therapy for AML with C-KIT tyrosine kinase domain mutation. JCB.

[23] Janghorban M, Farrell AS, Allen-Petersen BL, Pelz C, Daniel CJ, Oddo J, Langer EM, Christensen DJ, Sears RC (2014). Targeting c-MYC by antagonizing PP2A inhibitors in breast cancer. PNAS.

[24] Perrotti D, Neviani P (2013). Protein phosphatase 2A: a target for anticancer therapy. Lancet Oncol.

[25] Arriazu E, Pippa R, Odero MD (2016). Protein Phosphatase 2A as a Therapeutic Target in Acute Myeloid Leukemia. Front Oncol.

[26] Cristobal I, Blanco FJ, Garcia-Orti L, Marcotegui N, Vicente C, Rifon J, Novo FJ, Bandres E, Calasanz MJ, Bernabeu C, Odero MD (2010). SETBP1 overexpression is a novel leukemogenic mechanism that predicts adverse outcome in elderly patients with acute myeloid leukemia. Blood.

[27] Cristobal I, Rincon R, Manso R, Carames C, Zazo S, Madoz-Gurpide J, Rojo F, Garcia-Foncillas J (2015). Deregulation of the PP2A inhibitor SET shows promising therapeutic implications and determines poor clinical outcome in patients with metastatic colorectal cancer. Clin Cancer Res.

[28] Dong L, Zhu J, Wen X, Jiang T, Chen Y (2014). Involvement of SET in the Wnt signaling pathway and the development of human colorectal cancer. Oncol Lett.

[29] Hu X, Garcia C, Fazli L, Gleave M, Vitek MP, Jansen M, Christensen D, Mulholland DJ (2015). Inhibition of Pten deficient Castration Resistant Prostate Cancer by Targeting of the SET - PP2A Signaling axis. Sci Rep.

[30] Hung MH, Wang CY, Chen YL, Chu PY, Hsiao YJ, Tai WT, Chao TT, Yu HC, Shiau CW, Chen KF (2016). SET antagonist enhances the chemosensitivity of non-small cell lung cancer cells by reactivating protein phosphatase 2A. Oncotarget.

[31] Liu H, Gu Y, Wang H, Yin J, Zheng G, Zhang Z, Lu M, Wang C, He Z (2015). Overexpression of PP2A inhibitor SET oncoprotein is associated with tumor progression and poor prognosis in human non-small cell lung cancer. Oncotarget.

[32] Christensen DJ, Chen Y, Oddo J, Matta KM, Neil J, Davis ED, Volkheimer AD, Lanasa MC, Friedman DR, Goodman BK, Gockerman JP, Diehl LF, de Castro CM (2011). SET oncoprotein overexpression in B-cell chronic lymphocytic leukemia and non-Hodgkin lymphoma: a predictor of aggressive disease and a new treatment target. Blood.

[33] Christensen DJ, Ohkubo N, Oddo J, Van Kanegan MJ, Neil J, Li F, Colton CA, Vitek MP (2011). Apolipoprotein E and peptide mimetics modulate inflammation by binding the SET protein and activating protein phosphatase 2A. J Immunol.

[34] Agarwal A, MacKenzie RJ, Pippa R, Eide CA, Oddo J, Tyner JW, Sears R, Vitek MP, Odero MD, Christensen DJ, Druker BJ (2014). Antagonism of SET using OP449 enhances the efficacy of tyrosine kinase inhibitors and overcomes drug resistance in myeloid leukemia. Clin Cancer Res.

[35] Gusscott S, Jenkins CE, Lam SH, Giambra V, Pollak M, Weng AP (2016). IGF1R Derived PI3K/AKT Signaling Maintains Growth in a Subset of Human T-Cell Acute Lymphoblastic Leukemias. PloS one.

[36] Sanda T, Tyner JW, Gutierrez A, Ngo VN, Glover J, Chang BH, Yost A, Ma W, Fleischman AG, Zhou W, Yang Y, Kleppe M, Ahn Y (2013). TYK2-STAT1-BCL2 pathway dependence in T-cell acute lymphoblastic leukemia. Cancer Discov.

[37] You D, Xin J, Volk A, Wei W, Schmidt R, Scurti G, Nand S, Breuer EK, Kuo PC, Breslin P, Kini AR, Nishimura MI, Zeleznik-Le NJ (2015). FAK mediates a compensatory survival signal parallel to PI3K-AKT in PTEN-null T-ALL cells. Cell rep.

[38] Junttila MR, Puustinen P, Niemela M, Ahola R, Arnold H, Bottzauw T, Ala-aho R, Nielsen C, Ivaska J, Taya Y, Lu SL, Lin S, Chan EK (2007). CIP2A inhibits PP2A in human malignancies. Cell.

[39] Pippa R, Dominguez A, Malumbres R, Endo A, Arriazu E, Marcotegui N, Guruceaga E, Odero MD (2016). MYC-dependent recruitment of RUNX1 and GATA2 on the SET oncogene promoter enhances PP2A inactivation in acute myeloid leukemia. Oncotarget.

[40] Rao SS, O'Neil J, Liberator CD, Hardwick JS, Dai X, Zhang T, Tyminski E, Yuan J, Kohl NE, Richon VM, Van der Ploeg LH, Carroll PM, Draetta GF (2009). Inhibition of NOTCH signaling by gamma secretase inhibitor engages the RB pathway and elicits cell cycle exit in T-cell acute lymphoblastic leukemia cells. Cancer Res.

[41] Shepherd C, Banerjee L, Cheung CW, Mansour MR, Jenkinson S, Gale RE, Khwaja A (2013). PI3K/mTOR inhibition upregulates NOTCH-MYC signalling leading to an impaired cytotoxic response. Leukemia.

[42] Welcker M, Orian A, Jin J, Grim JA, Harper JW, Eisenman RN, Clurman BE (2004). The Fbw7 tumor suppressor regulates glycogen synthase kinase 3 phosphorylation-dependent c-Myc protein degradation. PANS.

[43] Faivre S, Djelloul S, Raymond E (2006). New paradigms in anticancer therapy: targeting multiple signaling pathways with kinase inhibitors. Semin Oncol.

[44] Ventura J-J, Nebreda ÁR (2006). Protein kinases and phosphatases as therapeutic targets in cancer. Clin Transl Oncol.

[45] Tyner JW, Yang WF, Bankhead A, Fan G, Fletcher LB, Bryant J, Glover JM, Chang BH, Spurgeon SE, Fleming WH, Kovacsovics T, Gotlib JR (2013). Kinase Pathway Dependence in Primary Human Leukemias Determined by Rapid Inhibitor Screening. Cancer Res.

[46] Porta C, Giglione P, Liguigli W, Paglino C (2015). Dovitinib (CHIR258, TKI258): structure, development and preclinical and clinical activity. Future Oncol.

[47] Scheid C, Reece D, Beksac M, Spencer A, Callander N, Sonneveld P, Kalimi G, Cai C, Shi M, Scott JW, Stewart AK (2015). Phase 2 study of dovitinib in patients with relapsed or refractory multiple myeloma with or without t(4;14) translocation. Eur J Haematol.

[48] Kim KB, Chesney J, Robinson D, Gardner H, Shi MM, Kirkwood JM (2011). Phase I/II and pharmacodynamic study of dovitinib (TKI258), an inhibitor of fibroblast growth factor receptors and VEGF receptors, in patients with advanced melanoma. Clin Cancer Res.

[49] Farrell AS, Pelz C, Wang X, Daniel CJ, Wang Z, Su Y, Janghorban M, Zhang X, Morgan C, Impey S, Sears RC (2013). Pin1 regulates the dynamics of c-Myc DNA binding to facilitate target gene regulation and oncogenesis. MCB.

[50] Lucas CM, Harris RJ, Giannoudis A, Clark RE (2015). c-Myc inhibition decreases CIP2A and reduces BCR-ABL1 tyrosine kinase activity in chronic myeloid leukemia. Haematologica.

[51] Hunger SP, Lu X, Devidas M, Camitta BM, Gaynon PS, Winick NJ, Reaman GH, Carroll WL (2012). Improved survival for children and adolescents with acute lymphoblastic leukemia between 1990 and 2005: a report from the children's oncology group. JOC.

[52] Weng AP, Ferrando AA, Lee W, Morris JP, Silverman LB, Sanchez-Irizarry C, Blacklow SC, Look AT, Aster JC (2004). Activating Mutations of NOTCH1 in Human T Cell Acute Lymphoblastic Leukemia. Science.

[53] Ferrando AA (2009). The role of NOTCH1 signaling in T-ALL. ASH Education Program Book.

[54] Bonnet M, Loosveld M, Montpellier B, Navarro JM, Quilichini B, Picard C, Di Cristofaro J, Bagnis C, Fossat C, Hernandez L, Mamessier E, Roulland S, Morgado E (2011). Posttranscriptional deregulation of MYC via PTEN constitutes a major alternative pathway of MYC activation in T-cell acute lymphoblastic leukemia. Blood.

[55] Chen YY, Hsieh CY, Jayakumar T, Lin KH, Chou DS, Lu WJ, Hsu MJ, Sheu JR (2014). Andrographolide induces vascular smooth muscle cell apoptosis through a SHP-1-PP2A-p38MAPK-p53 cascade. Sci Rep.

[56] O'Neil J, Grim J, Strack P, Rao S, Tibbitts D, Winter C, Hardwick J, Welcker M, Meijerink JP, Pieters R, Draetta G, Sears R, Clurman BE (2007). FBW7 mutations in leukemic cells mediate NOTCH pathway activation and resistance to gamma-secretase inhibitors. JEM.

[57] Gutierrez A, Pan L, Groen RW, Baleydier F, Kentsis A, Marineau J, Grebliunaite R, Kozakewich E, Reed C, Pflumio F, Poglio S, Uzan B, Clemons P (2014). Phenothiazines induce PP2A-mediated apoptosis in T cell acute lymphoblastic leukemia. JCI.

[58] Lou K-J (2014). Dual: Multi-Pronged Attack. Biocentury.

[59] Rhodes DR, Yu J, Shanker K, Deshpande N, Varambally R, Ghosh D, Barrette T, Pandey A, Chinnaiyan AM (2004). ONCOMINE: a cancer microarray database and integrated data-mining platform. Neoplasia.

