# Supplementary material for: Combined targeting of SET and tyrosine kinases provides an effective therapeutic approach in human T-cell acute lymphoblastic leukemia
Source: Oncotarget. 2016 Oct 1;7(51):84214–27. doi: 10.18632/oncotarget.12394 (PMC5356656; doi:10.18632/oncotarget.12394)
Supplement: Supplementary file 1 [file oncotarget-07-84214-s001.pdf]

Combined targeting of SET and tyrosine kinases provides an effective therapeutic approach in human T-cell acute lymphoblastic leukemia

Supplementary Materials

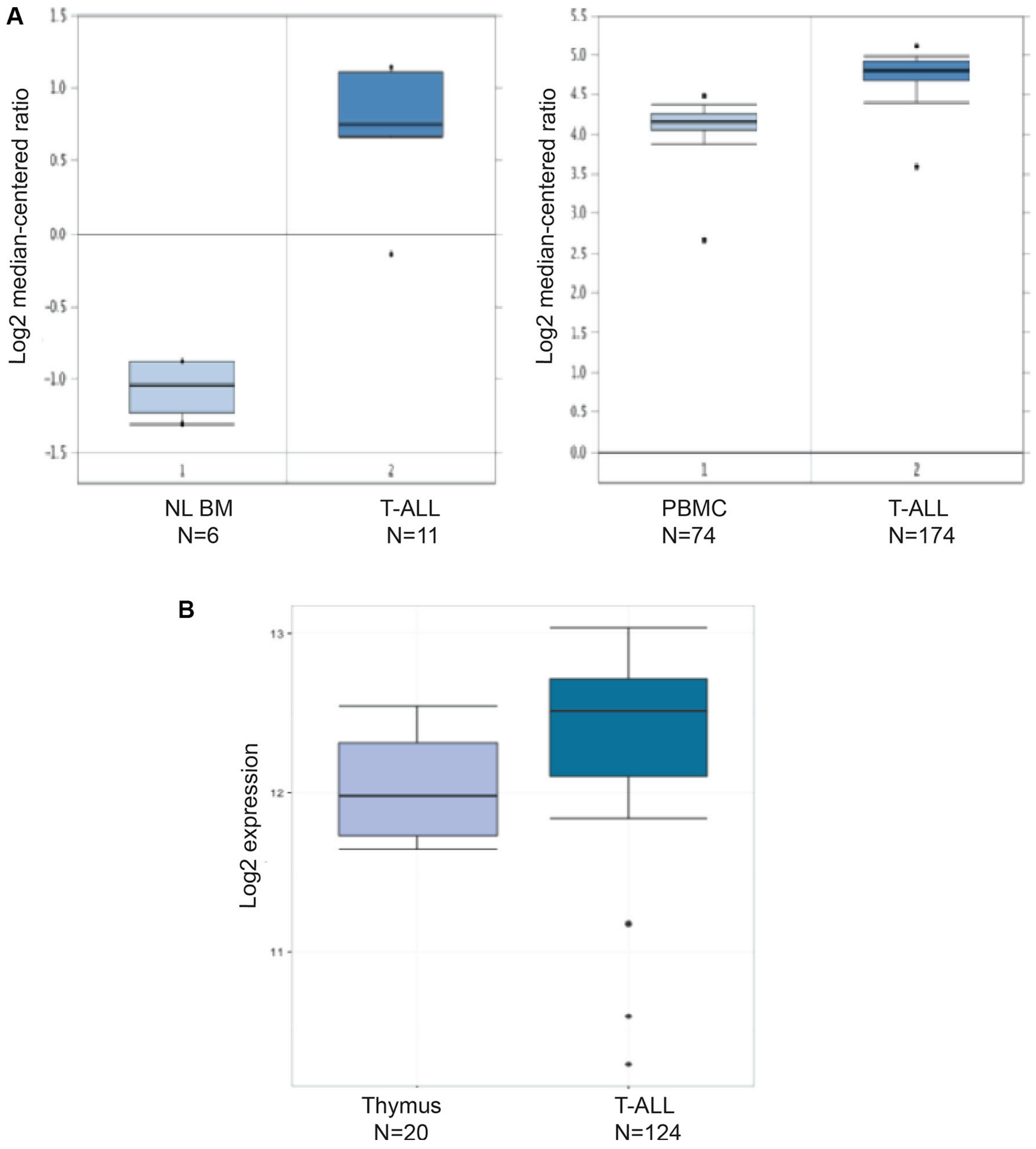

Supplementary Figure S1: SET is overexpressed in primary T-ALL samples. The expression of SET was compared between primary T-ALL and normal samples using available databases (**A**) Data from Oncomine (L) Andersson Leukemia Study (Expression is from Custom Array, Probe IMAGE:646801) (R) Haferlach Leukemia (Expression is from U133 Plus 2.0 Array, Probeset 40189_at). (**B**) Data is from GEO GSE46170 (Expression is from Affymetrix GeneChip HU-133 Plus.2, Probeset 40189_at). Note: Y-axis for Oncomine is their log2-median centered ratio. Y-axis for GEO public data is log2 expression.

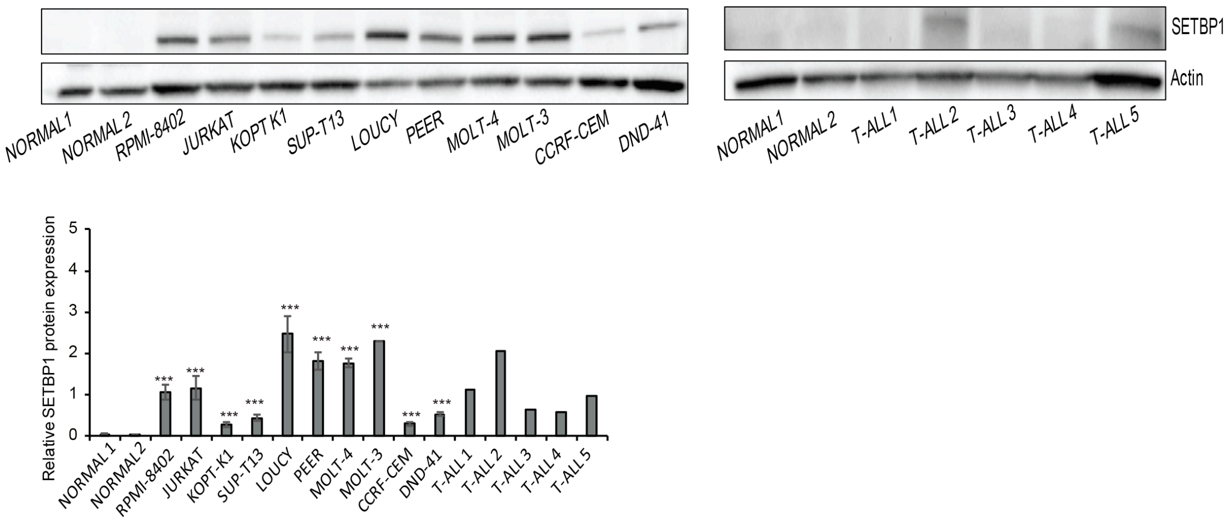

Supplementary Figure S2: (Related to Figure 1): SETBP1 is overexpressed in T-ALL cells. SETBP1 protein levels were tested by immunoblotting in T-ALL cell lines, primary T-ALL samples, and normal CD3+ cells. Protein levels were quantified by performing densitometric analysis of protein expression in each of the indicated samples and normalizing with total actin levels. The data represents mean+/−SEM from three independent experiments. $*p \leq 0.05$, $**p \leq 0.01$, $***p \leq 0.001$.

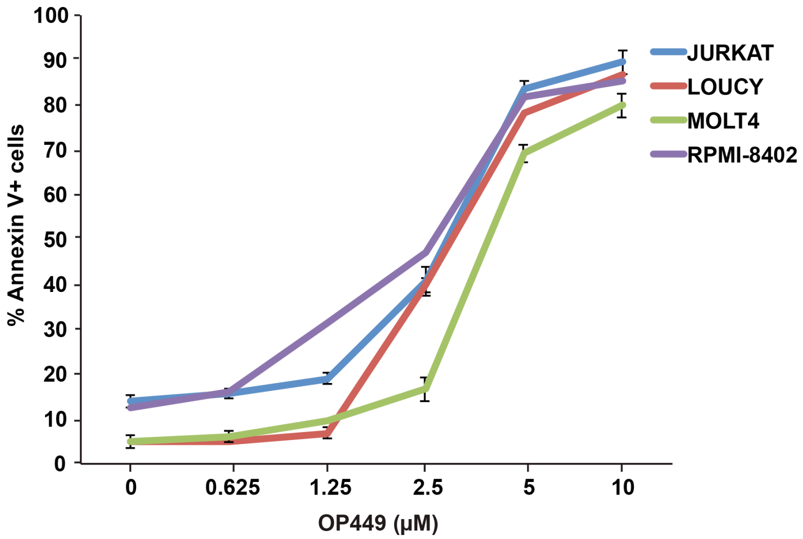

Supplementary Figure S3: OP449 inhibits growth of T-ALL cells by increasing apoptosis. T-ALL cell lines were cultured in graded concentrations of OP449. Cellular apoptosis was measured by annexin V staining at 48 hours. Results are graphed as the mean percent viability relative to untreated cells ± standard deviation.

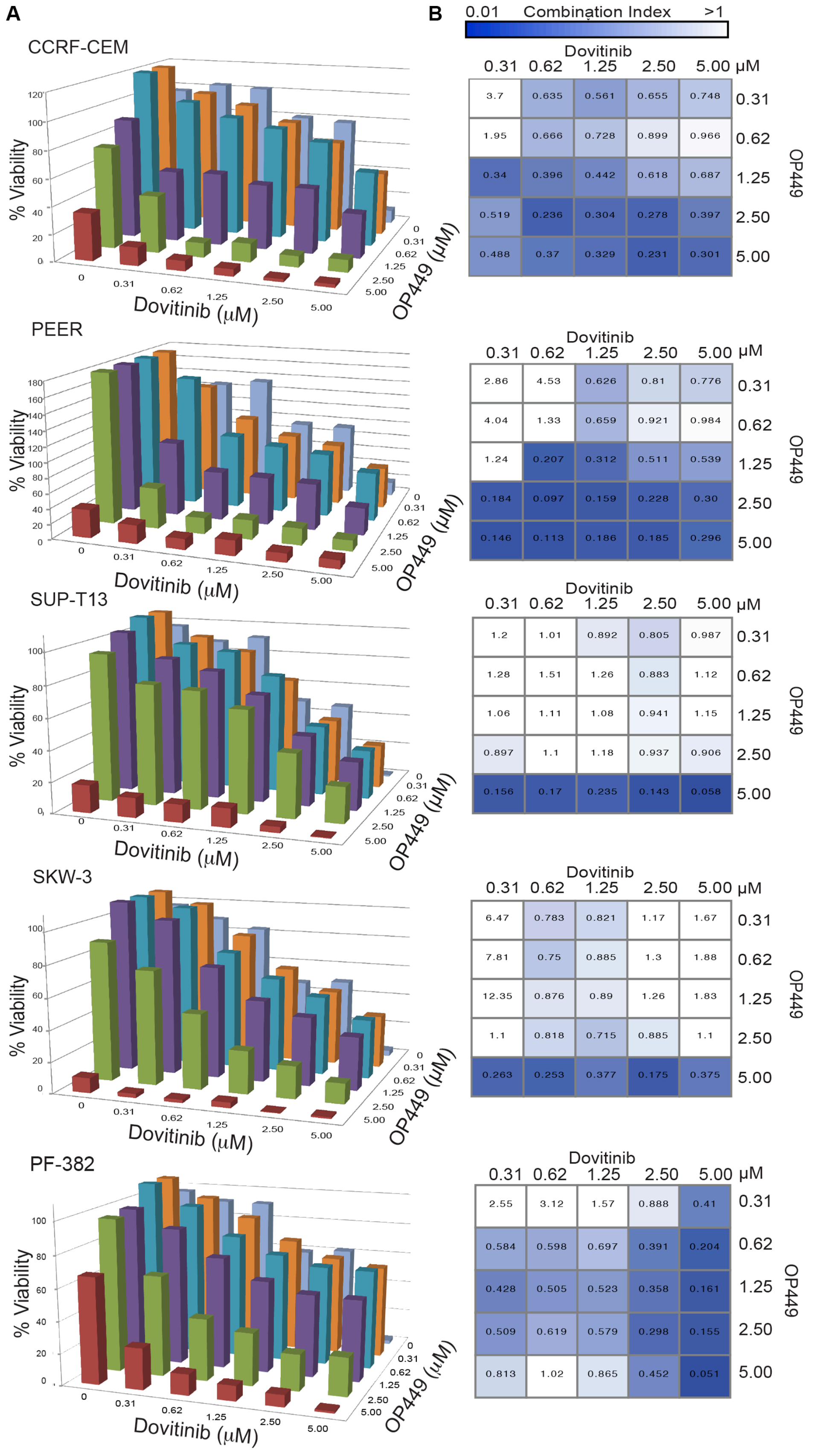

Supplementary Figure S4: Combination of OP449 and Dovitinib inhibits T-ALL growth synergistically. (**A**) T-ALL cell lines were cultured for 72 hours with indicated concentrations of OP449 or Dovitinib alone or combination. Cell viability was measured by MTS assay. (**B**) Heatmap of combination indices corresponding to A, where values less than one were considered synergistic.

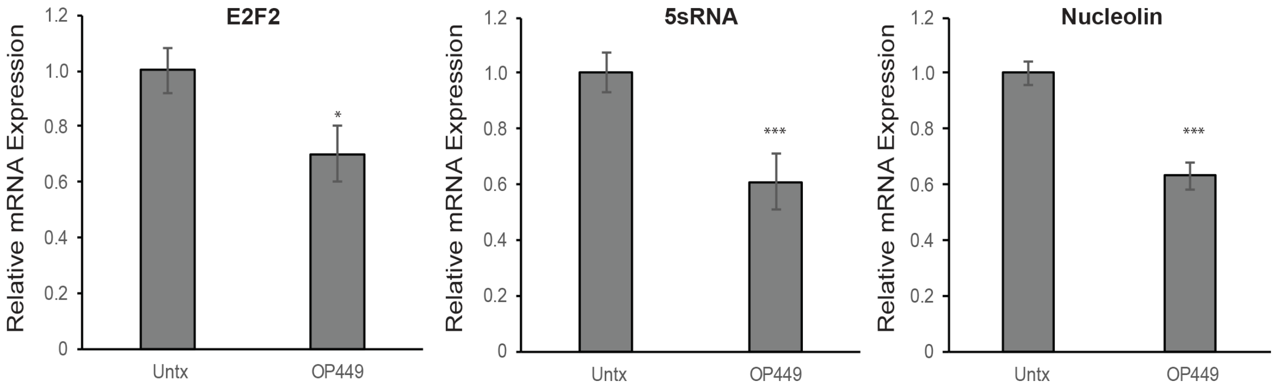

Supplementary Figure S5: SET antagonism inhibits the activity of c-MYC in T-ALL cells. RPMI-8402 T-ALL cell line was cultured for 48 hrs with 1.0 μM OP449 and the effect on c-MYC targets was measured by quantitative PCR analysis.

Supplementary Table S1: Characteristics of normal donors and T-ALL patient samples used for the study

Sample	Diagnosis	Gender	Age
Normal 1	Healthy Donor	Male	29
Normal 2	Healthy Donor	Female	24
Normal 3	Healthy Donor	N/A	N/A
Normal 4	Healthy Donor	N/A	N/A
Normal 5	Healthy Donor	N/A	N/A
T-ALL 1	T Lymphoblastic leukaemia/lymphoma	Male	65
T-ALL 2	T Lymphoblastic leukaemia/lymphoma	Male	16
T-ALL 3	T Lymphoblastic leukaemia/lymphoma	male	20
T-ALL 4	T Lymphoblastic leukaemia/lymphoma	Female	71
T-ALL 5	T Lymphoblastic leukaemia/lymphoma	Male	12

Supplementary Table S2: List of the Kinase inhibitors with relative IC50 values when used alone or in combination with OP449

Pathway tested	Small molecules tested	Small molecules tested as Single agent (IC_{50} in μM)	Small molecule inhiibitors + OP449 (IC_{50} in μM)
MULTI-KINASE	Dovitinib	1.29	0.92
SRC/ TEC/ TRK	GW-2580	10.0	10.0
JAK Family	Cytopia	10.0	10.0
PI3K/AKT	PI-103	1.0	1.2
MAPK	LY-333531	3.2	2.4
MAPK	JNK II	5.0	5.0
ERBB Family	Lapatinib	10.0	10.0
CDK	JNJ-7706621	3.8	4.4

The effect of OP449 on the growth of T-ALL cells in combination with various small molecule kinase inhibitors: Various agents representing each subclass of pathways were tested alone and in combination with OP449 to inhibit the growth of T-ALL cells using MOLT-4.
